# Interactive Effects of Water Deficiency and Endophytic *Beauveria bassiana* on Plant Growth, Nutrient Uptake, Secondary Metabolite Contents, and Antioxidant Activity of *Allium cepa* L.

**DOI:** 10.3390/jof8080874

**Published:** 2022-08-19

**Authors:** Lizeka Pretty Gana, Ninon G. E. R. Etsassala, Felix Nchu

**Affiliations:** Department of Horticultural Sciences, Faculty of Applied Sciences, Cape Peninsula University of Technology, P.O. Box 1905, Bellville 7535, South Africa

**Keywords:** antioxidant activities, *Allium cepa*, *B. bassiana*, water deficit, endophytic fungus, polyphenol and flavonol

## Abstract

The main aim of this research study was to assess the interactive effects of water deficiency and the inoculation of a growth medium with *Beauveria bassiana* on plant growth, nutrient uptake, secondary metabolite contents, and antioxidant capacity of *Allium cepa*. *A. cepa* seedlings were simultaneously exposed to one of three watering regime treatments (3-day, 5-day, and 7-day watering intervals) and *B. bassiana* or no-fungus treatment. While the longest watering interval induced reduced plant growth, plants inoculated with *B. bassiana* had better results than those in the no-fungus treatment. Significant interactive effects (DF = 2.0; *p* < 0.05) between fungus and the watering regime on P, K, and Fe contents were observed. Remarkably, at the 7-day watering interval, the polyphenol content (64.0 mg GAE/L) was significantly higher in the plants treated with *B. bassiana* than in the no-fungus-treated plants. The watering interval significantly affected (DF = 2, 6; F = 7.4; *p* < 0.05) total flavonol contents among the fungus-treated plants. The interaction of the watering interval and *B. bassiana* inoculation (DF = 2.0; F = 3.8; *p* < 0.05) significantly influenced the flavonol content in the onion bulbs and the antioxidant activities of onion bulbs in the FRAP assay (DF = 2.0; F = 4.1; *p* < 0.05).

## 1. Introduction

The rapidly growing demand for medicinal and nutraceutical plants is driving the search for more efficient strategies to cultivate medicinal and nutraceutical plants. Onion is one of the most consumed vegetables in the world [1]. However, because of increasing drought episodes worldwide and the high water demand for onions [2], it is necessary to develop efficient cultivation strategies that minimise water usage and optimise the plant’s nutraceutical qualities. According to Khokhar (2017), onions are more sensitive to water stress than other agronomic crops [3]. Soil cultivation is still the most popular approach for cultivating onions. However, soil cultivation has many challenges, including exposure to pests, high pesticides, and fertilisers [4]. Consequently, researchers are looking for other alternative cultivation methods that are more environmentally friendly and cost-effective. Hydroponics is a feasible, alternative approach for cultivating crops [5]. The biotic and abiotic factors can be manipulated in hydroponics to optimise plants’ secondary metabolite production and bioactivities [6]. Plants’ secondary metabolites not only play vital ecological roles in plants’ defense, protection, and signalling mechanisms [7], but are also exploited for their pharmaceutical attributes. To achieve the optimum cultivation of plants, it is essential to understand how plants respond to abiotic and biotic environmental changes for the optimal biosynthesis of medicinal and nutraceutical bioactive compounds.

Water stress is one of the most prominent abiotic environmental factors influencing plant growth and secondary metabolite contents [8,9]. It is a well-known limiting factor that affects various elements in plant growth and development [10]. Water stress can cause plant dehydration, stomatal closure, reduced gas exchanges, impairment of photosynthesis, and, ultimately, plant death [11]. However, plant survival varies with the plant species, growth stage, and the duration and severity of water deficiency [12]. Water shortage also decreases the total soil nutrient accessibility and root nutrient distribution [13]. However, water stress can also influence secondary metabolite contents in plants [14]. Indeed, many studies have revealed that water stress can induce higher concentrations of bioactive compounds in plants [9,15]. Results from a study on *Chrysanthemum morifolium* L. cultivars by Hodaei et al. [16] showed that the contents of six phenolic compounds such as chlorogenic acid, rutin, ferulic acid, quercetin, apigenin, and luteolin increased with increasing water stress, and cultivar and flavonoid gene–environment interactions influenced these responses.

The diverse roles of fungal entomopathogens, ubiquitous within the soil and susceptible insects, in host plant growth promotion and pest and pathogen management have sparked a great deal of interest in the application of fungal endophytes in crop cultivation [17]. The endophytic fungus–plant symbiotic relationship benefits the host plant and fungus [18,19]. Some entomopathogenic fungi, such as *Beauveria bassiana* and *Clonostachys rosea* (Hypocreales), can infect insects and colonise plant tissues as symptomless endophytes [20]. These fungi can live within plant tissues and proliferate under stressful environmental conditions, including drought [21]. They have been shown to produce bioactive compounds that promote plant resistance to pests and diseases and increase the rate of nutrient uptake, tolerance to abiotic and biotic stresses, and overall plant growth [18]. For example, in a recent study, entomopathogenic fungus *B. bassiana* optimised the yield and secondary metabolite contents of *A. cepa* plants in a hydroponic system [19]. Many studies have demonstrated that *B. bassiana* strains can potentially be employed to optimise the commercial production of crops [20].

The current study focuses on the combined effects of water stress and endophytic fungus colonisation on the plant growth, secondary metabolite production, and antioxidant activities of *A. cepa*, a member of the Allioideae subfamily and Amaryllidaceae family [21]. While many studies have separately investigated the effects of water, light, or nutrient stress and fungal endophytes on plant growth parameters and secondary metabolites, few studies have investigated the combined effects of abiotic and biotic factors in optimising the medicinal or nutraceutical properties of plants. The Allium genus comprises over 850 species, making it one of the largest monocot genera worldwide [22]. *Allium* species, such as onions, garlic, and leeks, are known for their medicinal and nutraceutical properties and have demonstrated substantial pharmaceutical and nutritional activities [23]. *Allium* spp. extracts contain several bioactive constituents, including phenolic compounds, organosulfur compounds, non-structural and soluble carbohydrates, organic acids, and various amino acids [24]. Phenolic compounds, including gallic acid, quercetin, coumaric acid, and ferulic acid, are found in *Allium* spp. [25,26]. Onions are rich sources of flavonoids and organosulfur compounds (allicin), both of which are potent antioxidants [27]. Hence, this investigation primarily aimed to determine the effects of combined water deficiency and the inoculation of growth medium with *B. bassiana* on plant growth, nutrient uptake, and secondary metabolite contents, as well as the antioxidant capacity of *A. cepa*. 

## 2. Materials and Methods

### 2.1. Plant Material

A total of 180 onion seedlings (Red Creole cultivar) sourced from a local retail nursery, Hart Nursery (PTY) Ltd. Ottery, Western Cape Province, South Africa, were selected for this experiment.

### 2.2. Preparation of Fungus (B. bassiana)

The fungal cultures of a *B. bassiana* strain (SM 3), which is currently being maintained at the Horticultural Department, Cape Peninsula University of Technology, were used in this study. The isolate was initially isolated from a local vineyard and was identified molecularly by Moloinyane and Nchu (2019) [28]. The fungus was cultured on a medium containing half-strength Potato Dextrose Agar (PDA) (19.5 g/1000 mL of water), 0.04 g streptomycin, and 0.02 g ampicillin sodium salt (Sigma-Aldrich (PTY) Ltd., Johannesburg, South Africa). The prepared medium was then transferred into Petri dishes with 9 and 14 cm diameters. Fungal cultures were incubated in the dark for three weeks at 25 ± 2 °C. Using a sterile spatula, the mature conidia of *B. bassiana* were scraped and transferred into a 50 mL centrifuge tube containing 30 mL of sterile distilled water (0.05% of Tween 80) and glass beads. The conidial suspension was vigorously shaken for three minutes, and after that, it was vortexed for another three minutes again using a vortex mixer at 3000 rpm to homogenise the conidial suspension. The homogeneous conidial suspension was transferred into 1000 mL bottles. The required conidia concentration of 1 × 10^6^ conidia mL^−1^ was determined using a hemocytometer. Sterile distilled water or conidial suspension was added when necessary to achieve the desired conidial concentration. A volume of 500 mL of conidial suspension was prepared in each 1000 mL bottle. 

### 2.3. Experimental Setup

The experiment was conducted in the greenhouse of the Department of Horticultural Sciences, Cape Peninsula University of Technology, Bellville Campus. The experiment ran for ten weeks from spring to early summer (October–December) at 28.9–36.9 °C and 51–59% RH. A completely randomised design was used to test the main effects of two factors (*B. bassiana* inoculation and watering regime) consisting of two *B. bassiana* treatments and three watering regimes on *A. cepa* plants. The effects of the interactions of the two factors on the plant growth, tissue nutrients and secondary metabolite contents, and antioxidant activity of the bulb extracts of onions were also assessed.

An inert substrate mix containing different substrates (silica sand, vermiculite, perlite, and coco peat) was mixed in a 1:1:1:1 ratio. Two-week-old seedlings were transplanted into the potted substrate mix individually in 15 cm diameter pots after rinsing under running tap water to remove all the potting soil around the roots. The seedlings were divided into two groups. One group was inoculated with *B. bassiana*; the second was not. Plants in both groups received water stress treatments, i.e., no watering for 3, 5, or 7 days. Thirty potted plants were randomly allocated and treated to either *B. bassiana* or no *B. bassiana* inoculation and one of three watering regimes (3-day, 5-day, and 7-day watering intervals). The watering interval was not extended beyond seven days because a pre-experimental investigation revealed that the plants wilted after seven days. In the fungus treatment, plant roots were inoculated with *B. bassiana* inoculum by drenching the substrate with 100 mL conidial suspension (1 × 10^6^ conidia mL^−1^) immediately after transplanting. *A. cepa* plants from each treatment were fed using hydroponics Nutrifeed fertilizer (Starke Ayres (PTY) Ltd., Cape Town, South Africa) consisting of the following ingredients: N (65 mg kg^−1^), P (27 mg kg^−1^), K (130 mg kg^−1^), Ca (70 mg kg^−1^), Cu (20 mg kg^−1^), Mo (10 mg kg^−1^), Fe (1500 mg kg^−1^), Mg (22 mg kg^−1^), S (75 mg kg^−1^), B (240 mg kg^−1^), Mn (240 mg kg^−1^), and Zn (240 mg kg^−1^). The fertiliser was mixed with deionised water at a dosage of 10 g/5 L. Each plant received 100 mL of the nutritional solution fortnightly. 

### 2.4. Plant Growth Parameters

After ten weeks post-treatment, i.e., ten weeks after the commencement of the experiment, the leaf length from the base of the leaf to the highest point was recorded using a 30 cm ruler for each replicate in the control and test treatments. *A. cepa* plants were uprooted, the bulb circumference was measured using a string (cm), and the bulbs’ fresh weights were recorded. Subsamples of 15 randomly selected *A. cepa* bulbs from the different treatments were then chopped, transferred into a paper bag, and oven-dried at 35 °C for two weeks. After that, the dried materials from each plant were weighed to obtain the bulb dry weight (g).

### 2.5. Colonisation of Tissues by Fungus

Four weeks after exposure of the experimental plants to the various treatments (watering regimes and *B. bassiana*), four leaf sections (1 × 1 cm^2^), carefully excised from four randomly selected plants in each treatment, were rinsed with sterile distilled water. They were then sterilised with 70% ethanol for 10 s and again rinsed with sterilised distilled water for 60 s. After that, they were placed on a Potato Dextrose Agar plate (PDA) containing half-strength Potato Dextrose Agar (PDA) (19.5 g/1000 mL), 0.04 g streptomycin, and 0.02 g ampicillin sodium salt. They were then incubated for 14 days in the dark at 25 °C.

### 2.6. Plant Tissue Nutrient Analyses

Bulbs of randomly selected plants from each treatment were analysed for macro-element and micro-element contents using an inductively coupled mass spectrometry (ICP-MS) and a LECO-nitrogen analyser with appropriate standards (Bemlab (PTY) Ltd., Somerset West, South Africa). Each treatment had three replicates. Briefly, the bulbs were cleaned in a Teepol solution, rinsed with deionised water, and dried in an oven at 70 °C overnight. Thereafter, the dried bulbs were pulverised, ashed at 480 °C, and agitated in a 50:50 HCl (50%) solution for extraction through filter paper [29], and then used to determine the potassium (K), phosphorus (P), calcium (Ca), magnesium (Mg), sodium (Na), manganese (Mn), iron (Fe), copper (Cu), zinc (Z), and boron (B) content of the extracts. Total combustion in a Leco N-analyser was used to assess the bulbs’ total nitrogen (N) contents. N, P, K, Ca, and Mg values were converted from percentages to mg/kg using a conversion factor of 10,000 [30].

### 2.7. Phytochemical Screening

After ten weeks in the glasshouse, three bulbs from each of the six treatments were randomly chosen and oven-dried for two weeks at 32 °C. A Jankel and Kunkel Model A 10 mill was used to grind each dry sample into a fine powder. The powdered material was labelled and packaged in sealable plastic bags.

#### 2.7.1. Total Alkaloid Contents

The total alkaloids in plant extracts were determined using the method given by Fadhil and Reza [31]. A quantity of 0.1 g of powdered bulb plant material was mixed with 20 mL of 60% ethanol and 40% distilled water in a centrifuge and left in the dark for 24 h. The sample’s absorbance at 417 nm was measured, and the concentration of mg atropine equivalent per g dry weight (mg AE/g DW) was estimated using an atropine 45 standard curve.

#### 2.7.2. Total Flavonoid Contents

The total flavonoid contents were determined using quercetin as a reference for 0, 5, 10, 20, 40, and 80 mg/L in 95% ethanol (Sigma-Aldrich (PTY) Ltd., Johannesburg, South Africa), as described by Daniels et al. [32]. The crude extract solution was made by combining 12.5 L of 0.1% Hydrochloric acid (HCl) (Merck (PTY) Ltd., Cape Town, South Africa) in 95% ethanol in the sample wells, then incubating at room temperature for 30 min. In the ethanol extracts, total flavonoid concentrations were reported as mg quercetin equivalent per gram dry weight (mg QE/g DW).

#### 2.7.3. Total Phenolic Contents

The total phenolic contents were determined using the Folin–Ciocalteau assay, as described by Singleton et al. [33]. Twenty-five grams of the material was mixed with 125 L of Folin–Ciocalteau reagent (1:10 dilution with distilled water) in a 96-well microplate (Merck (PTY) Ltd., Cape Town, South Africa). After 5 min, 100 L of the 7.5% Na_2_CO_3_ solution was added to each well’s mixture. The total phenolic contents of *A. cepa* dry bulb were measured in milligrams of gallic acid equivalents (GAE) per 100 g dry mass (mg GAE/100 g DW). All samples were carried out in duplicates.

### 2.8. Antioxidant Assays

#### 2.8.1. Ferric Reducing Antioxidant Power (FRAP) Assay

Benzie and Strain [34] describe a Ferric Reducing Antioxidant Power test comparable to the one employed here. The ferric tripyridyltriazine complex is reduced to its ferrous state in the presence of antioxidants in this test. The compounds used were as follows: 2.5 mL of a 10 mmol/L TPTZ (2, 4, 6-tripyridyl-s-triazine, Sigma) solution in 40 mmol/L HCl + 2.5 mL of 20 mmol/L FeCl_3_ and 25 mL of 0.3 mol/L acetate buffer, kept at pH 3.6 and heated at 37 °C. Forty microlitres of the sample supernatant was divided into aliquots and mixed with 0.2 mL distilled water and 1.8 mL FRAP reagent. After a 10 min incubation period at 37 °C, the absorbance of the reaction mixture was measured at 593 nm using a spectrophotometric technique. The standard solution was 1 mmol/L FeSO_4_, and the result was represented as the concentration of antioxidants with a ferric reducing capacity of 1 mmol/L FeSO_4_.

#### 2.8.2. Trolox Equivalent Antioxidant Capacity (TEAC) Assay

The antioxidant content in onion was measured using the TEAC method described by Miller et al. [35]. The antioxidant’s ability to scavenge the blue-green coloured ABTS^•+^ radical cation was compared to the water-soluble antioxidants’ ability to scavenge the blue-green coloured ABTS^•+^ radical cation.

### 2.9. Data Analysis

The experimental data (leaf height, fresh bulb weight, bulb circumference, dry bulb weight, and tissue nutrient contents) are presented as mean ± SE in tables. The data were analysed using one-way and two-way analyses of variance (ANOVA,) and the post-hoc Tukey HSD test was used to separate the means at a level of significance, *p* < 0.05. These computations were performed using STATISTICA software (TIBCO 1984–2018).

## 3. Results

### 3.1. Colonisation of Leaf Tissue

*Beauveria bassiana* was successfully re-isolated from the leaves of the *A. cepa* plants. All the fungus-treated plants recorded mycelial outgrowth on their leaf sections, representing 100% fungal colonisation. No fungal outgrowth occurred in the no-fungus plants.

### 3.2. Plant Growth Parameters

#### 3.2.1. Leaf Length

At ten weeks post-treatment, fungus-treated plants had significantly (DF = 1, 58; *p* < 0.01) longer leaves than the no-fungus plants; this was the same for both plants exposed to the 3-day and 5-day watering intervals. However, there was no significant difference (DF = 1, 58; F = 1.5; *p* > 0.05) in the leaf length of *A. cepa* plants exposed to the 7-day watering regime between the fungus-treated plants and the no-fungus plants. Generally, longer leaves were associated with a shorter watering interval and *B. bassiana* inoculation. The interaction between the fungus and watering regime on leaf height of *A. cepa* was significant (DF = 2; F = 22.3; *p* < 0.05).

#### 3.2.2. Bulb Circumference

There was no significant difference (DF = 1, 30; F = 2.2; *p* = 0.1) in the bulb circumference of the fungus and no-fungus plants exposed to low water stress (3-day watering interval) ten weeks post-treatment. However, the fungus treatment produced larger bulbs (8.8 ± 0.2 cm) than those with no fungus (8.2 ± 0.4 cm). Although the bulb circumferences varied significantly (DF = 1, 30; *p* < 0.05) between the fungus and the no-fungus treatments at the 5-day and 7-day watering intervals, there were no significant interactive effects (DF = 2.0; F = 2.6; *p* > 0.05) on fungus and watering regime.

#### 3.2.3. Bulb Wet Weight

The wet weights of the onion bulbs varied significantly (DF = 1, 30; *p* < 0.05) between fungus and no-fungus treatments at the different watering regime treatments. Fungus-treated plants watered at 3-day intervals had the highest mean (13.4 ± 0.7 g) bulb wet weight. There was a significant interactive effect (DF = 2.0; F = 22.3; *p* < 0.05) between fungus and watering regimes. 

#### 3.2.4. Bulb Dry Weight

At ten weeks post-treatment, there was no marked significant difference (DF = 1, 28; F = 3.4, *p* > 0.05) in the bulb dry weights of *A. cepa* plants exposed to the 3-day watering regime in the fungus and no-fungus treatments, ten weeks post-treatment (Table 1). There was a significant difference (DF = 1, 28; *p* < 0.05) in the bulb dry weight of *A. cepa* plants exposed to 5-day and 7-day watering regimes at ten weeks post-treatment (Table 1), with the fungus-treated plants having heavier bulbs than the no-fungus plants. Based on a two-way ANOVA, the interactive effect of watering regime and fungus (*B. bassiana* inoculum) on bulb dry weight was not significant (DF = 2.0; F = 0.9; *p* > 0.05).

### 3.3. Bulb Tissue Analysis

#### 3.3.1. Macronutrients 

There was no significant difference in the *A. cepa* plant tissue levels of C, N, P, K, Ca, and Mg on plants exposed to the 3-day watering regime (DF = 1, 4; *p* > 0.05), as shown in Table 2. This was the same for the 7-day watering regime, except for the mean Ca level in the fungus-treated plants, which was significantly higher compared with the no-fungus treatment (DF = 1, 4; F = 11.0; *p* < 0.05). Interestingly, there was a significant difference (DF = 1, 4; *p* < 0.05) in the bulb tissue levels of C, P, K, Ca, and Mg (DF = 1, 4; *p* < 0.05) in plants exposed to 5-day watering regimes, and higher concentrations of C, P, Ca, and Mg occurred in the fungus-treated plants. However, the N levels in the bulb tissue were not significantly different (DF = 1, 4; F = 0.0; *p* > 0.05) between plants in the fungus and no-fungus treatment at the different watering regimes. Significant interactive effects (DF = 2.0; *p* < 0.05) between fungus and watering regimes on P and K were observed. 

#### 3.3.2. Micronutrients 

After ten weeks post-treatment, there was no significant difference between the fungus-treated and no-fungus plants in Na, Fe, and Cu levels at the 3-day watering regime (DF = 1, 4; *p* > 0.05); however, the plant tissue levels of Mn and Zn in the bulbs varied significantly (DF = 1, 6; *p* < 0.05) with watering regimes among the fungus and no-fungus plants (Table 3). On the 3-day and 5-day watering regimes, the tissue nutrient uptake between the fungus-treated and no-fungus plants was significantly different only in the Zn levels (DF = 1, 4; F = 8.3, *p* < 0.05). The 7-day watering regime did not significantly differ in the tissues Na, Mn, Fe, Cu, and Zn (DF = 1, 4; *p* > 0.05) (Table 3). The interactive effect between fungus and watering regime on tissue Fe level was significant (DF = 2.0; *p* < 0.05). 

### 3.4. Phytochemical Contents

Alkaloids were not detected in this experiment in all the watering regimes and fungus treatments. There was no significant difference in the total polyphenol contents of plants at all the watering intervals (DF = 1, 4; *p* > 0.05) when the fungus and no-fungus treatments were compared (Table 4). Remarkably, at the 7-day watering interval, the polyphenol content (64.0 mg GAE/L) was significantly higher in plants treated with *B. bassiana* than in the no-fungus plants. Overall, however, the interaction of watering interval and fungus did not significantly (DF = 2.0; F = 3.4; *p* > 0.05) influence polyphenol contents in the onion bulbs based on a two-way ANOVA.

Generally, there was no significant difference in flavonol contents (DF = 1, 4; *p* > 0.05) between the plants inoculated with the fungus and those with no fungus at all three watering regimes (3-day, 5-day, and 7-day). However, the watering interval significantly affected (DF = 2, 6; F = 7.4; *p* < 0.05) total flavonol contents among the fungus-treated plants; the flavonol contents increased with the length of the watering interval in the fungus-treated plants. Noticeably, *B. bassiana* inoculation favoured a significantly higher accumulation of polyphenols in the onion bulbs at the 7-day watering interval compared to the no-fungus treatment (DF = 1, 4; *p* < 0.05); this is a reversal of the results obtained in the 3-day and 5-day treatments. The interaction of watering interval and fungus (DF = 2.0; F = 3.8; *p* < 0.05) significantly influenced flavonol contents in the onion bulbs, based on a two-way analysis of variance.

### 3.5. Antioxidant Activity

Interestingly, there were significant differences among plants exposed to the 7-day watering regime (most extended watering interval) (DF = 1, 4; F = 11.8; *p* < 0.05) in the FRAP and TEAC bioassays (Table 5), with the *B. bassiana*-treated plants yielding consistently higher antioxidant activity. Based on a two-way ANOVA, the interaction between water intervals and inoculation with *B. bassiana* significantly (DF = 2.0; *p* < 0.05) influenced the antioxidant activities of onion bulbs in the FRAP and TEAC bioassays. 

## 4. Discussion

Water is essential for plant growth and survival. Depleting and disappearing freshwater resources, driven mainly by over-exploitation, pollution, and land-use changes, have led to a drastic scarcity of agricultural water. The fresh-water shortage, therefore, constitutes a danger to agriculture’s long-term viability [8,36,37]. Water deficiency stress is an abiotic factor that impacts plant growth and is one of the most growth-limiting factors [38]. In the current study, the results showed that exposing onions to a short watering interval and *B. bassiana* inoculation enhanced plant growth, whereas increasing the watering intervals from 3-days to 7-days consistently improved secondary metabolite contents and antioxidant activities among plants exposed to *B. bassiana* inoculum. However, the interactive effects of endophytic *B. bassiana* and watering regime on growth parameters, secondary metabolite contents, and antioxidant activities varied.

Water in a plant growth medium dissolves critical minerals and nutrients that are absorbed through the roots of plants. Hence, the higher growth obtained among plants in the 3-day watering interval than in the 5-day and 7-day intervals is expected. Previously, McDowell et al. (2011) [39] and Breda et al. (2006) [40] reported that water stress is a hindrance to plant growth and development because it restricts access to the resources needed for photosynthesis owing to stomatal closure and reduced internal water. Interestingly, although the growth results showed reductions in all growth parameters assessed, the reductions in growth were much more pronounced among plants that were not inoculated with *B. bassiana*. Prior studies reported that fungi that live as endophytes in host plants have plant growth-promoting properties [41,42]. Numerous studies have shown that when used as an endophyte in agricultural plants, entomopathogenic fungi can enhance plant height, weight, and other growth indices [42,43,44,45]. For example, endophytic *B. bassiana* and *Purpureocillium lilacinum* enhanced the growth and dry biomass of cotton plants [44].

Another mechanism through which endophytic fungi alleviate environmental stress on their hosts is by increasing mineral uptake. For example, García-Latorre et al. (2021) [46] demonstrated that *Sporormiella intermedia* increased the mineral uptake of Ca, Cu, Mn, Pb, Tl, and Zn in *Trifolium subterraneum* (subclover); and *Mucor hiemalis* increased the uptake of K and Sr in *Poa pratensis* (Kentucky bluegrass). In the current study, phosphorus levels in the bulbs were significantly higher in the fungus-treated plants than in the no-fungus plants at the 5-day watering interval. Moreover, *B. bassiana* inoculation and watering intervals had a significant interactive effect on P and K levels. Although the K level in the fungus-treated plants was lower than in plants in the no-fungus treatment, at the most extended watering interval (7 days), Ca, Mg, P, and N levels were more elevated in the fungus-treated plants, corroborating the argument that endophytic fungi alleviate the negative impacts of water stress by enhancing the uptake of essential macronutrients. These nutrients are essential for disease resistance, photosynthesis, cell membrane integrity, protein synthesis, resistance to abiotic and biotic stresses, pollen production, and enhancement of antioxidant enzymes and chlorophyll levels in plant tissues [47].

The mechanisms by which fungal endophytes induce water and nutrient uptake by plant hosts include increased production of phytohormones that increase root biomass; improved fungal colonisation of roots and hyphal interception of nutrients; enhanced secretion of hydrolytic enzymes that enhance nutrient solubilisation; increased activity of plasma membrane-associated proton-pumps, and elevated expression of phosphate transporters; and enhanced siderophore binding sites that chelate Fe^3+^ ions and bind to Cu^2+^, Zn^2+^, and Mn^3+^ [47,48,49]. Interestingly, a significant interactive effect of watering interval and *B. bassiana* inoculation on tissue Fe content was observed in this study, with the fungus-treated plants having higher Fe content at 5-day and 7-day watering intervals. The Mn and Zn levels in plants varied significantly between the fungal treatments, with the fungus-treated plants recording higher Mn than the no-fungus plants at the 3-day watering interval, while Zn content was higher in the no-fungus plants than fungus-treated plants at the 5-day watering interval.

When the watering interval was increased from 3 days to 7 days, the secondary metabolite contents also increased. Remarkably, the highest total polyphenol (64.0 ± 6.4 mg GAE/L) and flavonol (25.3 ± 4.5 mg QE/L) contents were obtained in plants exposed to both a 7-day watering regime and *B. bassiana* treatments. The results showed that the flavonol level in onion bulbs was enhanced when the watering interval was extended to 7 days, and *B. bassiana* inoculation and a long watering interval also enhanced the total polyphenol contents. Subjecting plants to reduced water can increase carbon-based secondary metabolites [50]. Plants employ adaptive physiological responses to cope with the environmental stress elicitors; for example, plants may produce chemotypes such as lipoic and ascorbic acid, flavonoids (quercetin), carotenoids, arylamines, aliphatic and unsaturated fatty acids as part of their chemical defense response to stress [14]. However, water stress can have varied effects on plants’ production of secondary metabolites; for example, artemisinin, arteannuin-B, artemisinic acid, and dihydroartemisinic acid, major constituents of *Artemisia annua* L., were negatively influenced by water deficit stress, while sesquiterpenes and other low molecular weight volatiles were positively impacted by water deficits [51]. Roos and Nchu (2021) [9] reported that subjecting *Salvia* species to different water stress levels influenced the accumulation of secondary metabolites.

Endophytic fungi are a well-known source of bioactive secondary metabolites [52]. Their presence in plants could also induce plants to produce more secondary metabolites, conferring protection against biotic and abiotic stress factors. During the symbiotic interaction between a host plant and an endophyte, both organisms could engage in horizontal gene transfer and the synthesis, accumulation, and transfer of common metabolites to their corresponding symbiotic systems [53]. Endophytes, including fungal endophytes, can produce siderophores, which chelate the ferric iron from a plant growth medium and make it available for microbial and plant cells [54]. The current results showed higher levels of Fe (407.5 ± 114.0 mg/kg) in the fungus-treated plants.

Antioxidant activities increased most in bulbs exposed to fungus and the most extended watering interval. This finding suggests that *B. bassiana* inoculation enhances antioxidant activities, especially at the long watering interval. These results also correlate with the level of total phenolic contents. Flavonols and phenolic compounds have antioxidant activities [55]. In addition, the antioxidant activities were associated with the higher P, N, Mg, Ca, and Fe content in this study. These elements are essentials in plant physiological functions and the synthesis of primary metabolites. Some primary metabolites are transformed into intermediary compounds that act as precursors for synthesising carbon-based secondary metabolites by plants [56].

## 5. Conclusions

The key findings are that endophytic *B. bassiana* inoculation not only alleviates the adverse effects caused by water deficits on plant growth but also synergistically interacts with the most extended watering interval (7-day) to enhance total polyphenol and flavonol contents as well as the antioxidant activities of onion bulbs. The benefit of *B. bassiana* inoculation in improving the synthesis of secondary metabolites and antioxidant activities becomes evident under a high water deficit condition. Furthermore, the results show that the action of the *B. bassiana* fungus on growth and secondary metabolites is potentially mediated by the enhanced acquisition of essential nutrients such as the P, Ca, Mg, and Fe levels. However, it is necessary to study the role of phytohormones in mediating the growth-promoting effects of the fungus under water stress conditions. In conclusion, incorporating endophytic *B. bassiana* could improve the nutraceutical value of onions and reduce yield losses in water-scarce areas.

## Figures and Tables

**Table 1:** Effects of the watering regime and endophytic *B. bassiana* on plant growth parameters (mean ± SE) of *A. cepa*.

Watering Interval	Leaf Length (cm)		Bulb Circumference (cm)		Bulb Wet Weight (g)		Bulb Dry Weight (g)	
	Fungus	No Fungus	Fungus	No Fungus	Fungus	No Fungus	Fungus	No Fungus
3-day	60.8 ± 0.4 a (A)	44.4 ± 1.0 b (A)	8.8 ± 0.2 a (A)	8.2 ± 0.4 a (A)	13.4 ± 0.7 a (A)	10.4 ± 0.8 b (A)	2.3 ± 0.1 a (A)	1.9 ± 0.2 a (A)
5-day	48.3 ± 0.83 a (B)	41.8 ± 1.8 b (AB)	6.8 ± 0.2 a (B)	5.0 ± 0.2 b (B)	7.5 ± 0.4 a (B)	3.7 ± 0.2 b (B)	1.2 ± 0.1 a (B)	0.7 ± 0.1 b (B)
7-day	41.2 ± 1.0 a (C)	39.2 ± 1.2 a (B)	6.5 ± 0.2 a (B)	5.3 ± 0.2 b (B)	6.9 ± 0.4 a (B)	4.4 ± 0.4 b (B)	1.2 ± 0.1 a (B)	0.8 ± 0.1 b (B)
Two-way ANOVA	DF = 2.0; F = 22.3; *p* < 0.05		DF = 2.0; F = 2.6; *p* > 0.05		DF = 2.0; F = 22.3; *p* < 0.05		DF = 2.0; F = 0.2; *p* > 0.05	

Means followed by the same lowercase letters in the same row are not significantly different following Tukey’s test (*p* > 0.05). Means followed by the same uppercase letters in the same column are not significantly different following Tukey’s test (*p* > 0.05). DF: degree of freedom; F: F value.

**Table 2:** Effects of water deficiency and the inoculation of growth medium with *B. bassiana* on the macronutrient content in bulbs of *A. cepa*.

	C (mg/kg)		N (mg/kg)		P (mg/kg)		K (mg/kg)		Ca (mg/kg)		Mg (mg/kg)	
Watering Interval	Fungus	No Fungus	Fungus	No Fungus	Fungus	No Fungus	Fungus	No Fungus	Fungus	No Fungus	Fungus	No Fungus
3-day	473,350.0 ± 5686.9 a (A)	382,416.7 ± 77,822.7 a (A)	28,900.0 ± 866.0 a (A)	31,100.0 ± 288.7 a (A)	450.0 ± 28.9 a (B)	400.0 ± 0.0 a (B)	13,450.0 ± 28.9 a (A)	9350.0 ± 1587.7 a (B)	11,800.0 ± 288.7 a (B)	9000.0 ± 1154.7 a (A)	4300.0 ± 173.2 a(A)	3750.0 ± 721.9 a (A)
5-day	461,350.0 ± 2280.5 a (A)	453,950.0 ± 664.0 b (A)	27,250.0 ± 433.0 a (A)	27,450.0 ± 1010.4 a (B)	700.0 ± 0.0 a (A)	400.0 ± 0.0 b (B)	14,250.0 ± 490.8 a (A)	10,850.0 ± 606.2 b (AB)	14,200.0 ± 519.6 a (A)	11,100.0 ± 288.7 b (A)	5550.0 ± 86.6 a (A)	4150.0 ± 144.3 b (A)
7-day	459,050.0 ± 1760.9 a (A)	472,850.0 ± 17,176.1 a (A)	26,750.0 ± 1587.7 a (A)	26,050.0 ± 202.0 a (B)	550.0 ± 28.9 a (B)	516.7 ± 44.1 a (A)	13,100.0 ± 12.47 a (A)	16,000.0 ± 1674.3 a (A)	11,800.0 ± 519.6 a (B)	10,050.0 ± 86.6 b (A)	4700.0 ± 519.6 a (A)	4150.0 ± 86.6 a (A)
Two-way ANOVA	DF = 2.0; F = 1.4; *p* > 0.05		DF = 2.00; F = 1.4; *p* > 0.05		DF = 2.0; F = 37.5; *p* < 0.05		DF = 2.0; F = 7.5; *p* < 0.05		DF = 2.0; F = 0.7; *p* > 0.05		DF = 2.0; F = 0.8; *p* > 0.05	

Means followed by the same lowercase letters in the same row are not significantly different following Tukey’s test (*p* > 0.05). Means followed by the same uppercase letters in the same column are not significantly different following Tukey’s test (*p* > 0.05).

**Table 3:** Effects of water deficiency and the inoculation of growth medium with *B. bassiana* on micronutrient content in leaves of *A. cepa*.

	Na (mg/kg)		Mn (mg/kg)		Fe (mg/kg)		Cu (mg/kg)		Zn (mg/kg)	
Watering Interval	Fungus	No Fungus	Fungus	No Fungus	Fungus	No Fungus	Fungus	No Fungus	Fungus	No Fungus
3-day	688. 5 ± 32.0 a (A)	665.5 ± 101.3 a (A)	32.8 ± 0.3 a (A)	25.8 ± 1.2 b (A)	251.5 ± 21.7 a (A)	352.0 ± 101.6 a (A)	3.0 ± 0.23 a (A)	3.5 ± 0.40 a (A)	6.3 ± 0.63 b (A)	8.1 ± 0.1 a (B)
5-day	1031.0 ± 120.67 a (A)	1286.7 ± 330.97 a (A)	45.8 ± 1.62 a (A)	43.5 ± 3.41 a (B)	321.0 ± 74.48 a (A)	122.1 ± 35.19 a (A)	3.0 ± 0.1 a (A)	2.4 ± 0.4 a (A)	10.2 ± 1.6 b (A)	19.9 ± 2.9 a (A)
7-day	941.5 ± 103.1 a (A)	1076 ± 112.0 a (A)	38.85 ± 8.4 a (A)	43.3 ± 0.5 a (B)	407.5 ± 114.0 a (A)	124.0 ± 6.9 a (A)	2.8 ± 0.14 a(A)	2.9 ± 0.3 a (A)	9.3 ± 2.1 a (A)	5.1 ± 1.2 a (AB)
Two-way ANOVA	DF = 2.0; F = 0.4; *p* > 0.05		DF = 2.0; F = 1.1; *p* > 0.05		DF = 2.0; F = 4; *p* < 0.05		DF = 2.0; F = 2.0; *p* > 0.05		DF = 2.0; F = 2.6; *p* > 0.05	

Means followed by the same lowercase letters in the same row are not significantly different following Tukey’s test (*p* > 0.05). Means followed by the same uppercase letters in the same column are not significantly different following Tukey’s test (*p* > 0.05).

**Table 4:** The effects of *B. bassiana* inoculation and watering intervals on the secondary metabolite contents of *A. cepa*.

Secondary Metabolites				
	Total Polyphenol (mg GAE/L)		Total Flavonol (mg QE/L)	
Watering Interval	Fungus	No Fungus	Fungus	No Fungus
3-day	27.5 ± 6.3 a (A)	30.1 ± 3.2 a (A)	7.4 ± 0.8 a (B)	10.7 ± 1.4 a (A)
5-day	42.2 ± 12.7 a (A)	46.0 ± 2.3 a (A)	11.4 ± 3.9 a (AB)	13.8 ± 2.0 a (A)
7-day	64.0 ± 6.4 a (A)	35.7 ± 5.7 b (A)	25.3 ± 4.5 a (A)	14.6 ± 2.6 a (A)
Two-way ANOVA	DF = 2.0; F = 3.4; *p* > 0.05		DF = 2.0; F = 3.8; *p* < 0.05	

Means followed by the same lowercase letters in the same row are not significantly different following Tukey’s test (*p* > 0.05). Means followed by the same uppercase letters in the same column are not significantly different following Tukey’s test (*p* > 0.05).

**Table 5:** The effects of *B. bassiana* inoculation and watering intervals on the antioxidant capacity of *A. cepa*.

	FRAP (umol AAE/L)		TEAC (umol TE/L)	
Watering Interval	Fungus	No Fungus	Fungus	No Fungus
3-day	283.1 ± 30.4 a (A)	291.7 ± 13.8 a (A)	142.9 ± 15.4 a (B)	170.5 ± 7.8 a (B)
5-day	343.9 ± 71.1 a (A)	333.4 ± 17.4 a (A)	195.8 ± 29.9 a (AB)	220.8 ± 14.2 a (A)
7-day	492.2 ± 54.5 a (A)	289.8 ± 22.0 b (A)	273.5 ± 15.0 a (A)	171.6 ± 8.8 b (B)
Two-way ANOVA	DF = 2.0; F = 4.1; *p* < 0.05		DF = 2.00; F = 9.7; *p* < 0.05	

Means followed by the same lowercase letters in the same row indicate no significant difference between fungus and no fungus treatment following Tukey’s test (*p* > 0.05). Means followed by the same uppercase letters in the same column are not significantly different following Tukey’s test (*p* > 0.05).
